# Polymer Inclusion Membranes (PIMs) Doped with Alkylimidazole and their Application in the Separation of Non-Ferrous Metal Ions

**DOI:** 10.3390/polym11111780

**Published:** 2019-10-30

**Authors:** Elzbieta Radzyminska-Lenarcik, Malgorzata Ulewicz

**Affiliations:** 1 Faculty of Chemical Technology and Engineering, UTP University of Science and Technology, Seminaryjna 3, PL 85-326 Bydgoszcz, Poland; 2 Czestochowa University of Technology, Dabrowskiego 69 Street, PL 42-201 Czestochowa, Poland; ulewicz@bud.pcz.pl

**Keywords:** polymer inclusion membrane, metal separation, imidazole derivatives

## Abstract

The study involved the transport of zinc(II), cadmium(II), and nickel(II) ions from acidic aqueous solutions using polymer inclusion membranes (PIMs). PIMs consisted of cellulose triacetate (CTA) as a support; o-nitrophenyl pentyl ether (o-NPPE) as a plasticizer; and 1-octylimidazole (**1**), 1-octyl-2-methylimidazole (**2**), 1-octyl-4-methylimidazole (**3**), or 1-octyl-2,4-dimethylimidazole (**4**) as ion carriers. The membranes were characterized by means of atomic force microscopy (AFM) and scanning electron microscopy (SEM). The results show that Zn(II) and Cd(II) are effectively transported across PIMs, while Ni(II) transport is not effective. The rate of transport of metal ions across PIMs is determined by the diffusion rate of the M(II)–carrier complex across the membrane. The best result achieved for Zn(II) removal after 24 h was 95.5% for the ternary Zn(II)–Cd(II)–Ni(II) solution for PIM doped (**4**). For this membrane, the separation coefficients for Zn(II)/Cd(II), Zn(II)/Ni(II), and Cd(II)/Ni(II) were 2.8, 104.5, and 23.5, respectively. Additionally, the influence of basicity and structure of carrier molecules on transport kinetics was discussed.

## 1. Introduction

Zinc, cadmium, and nickel are among the most commonly used metals in industries. Due to their wide application, zinc, nickel, and cadmium are classified as strategic metals [1,2]. They are used in various branches of industry and in new fields of technology. The wide industrial use of those metals causes the generation of metal-bearing wastes in the form of sewage, dust, sludge, waste heaps, etc. [3,4,5]. An increasing interest in and consumption of these metals results in an increase of their prices, as well as a growing interest even in poor raw materials for their production. Therefore, the metal bearing waste has become an increasingly demanded raw material, and methods for reclaiming waste from industrial waste are steadily gaining in importance. During the last fifty years, solvent extraction has been a technique widely used for the processing of metal-bearing poor raw materials [6,7,8,9].

This technique has frequently been used in the extraction of some non-ferrous metals [10,11,12,13,14,15,16,17]. An increasing demand for metal production has led to the search for more efficient and economical methods required by the industry for purification of wastes. Membrane technologies have become an important alternative to conventional processes employed for wastewater treatment, and separation and recovery of target metals [17,18,19,20,21]. The selective transport of metal ions has been widely studied with supported (SLMs) and polymer inclusion (PIMs) liquid membranes [22,23,24,25,26,27,28,29,30,31,32,33]. Their high selectivity, high diffusion rates, and the possibility to concentrate ions have made them particularly useful [20,34].

The microstructure of the membrane surface is one of the important aspects influencing the process of metal ions separation. The supported liquid membranes (SMLs) are characterized by a structure strictly determined by the use of the specific polymer film (e.g., polyamide, Teflon). The commercially available membrane sheets (e.g., Celgard 2400, Accurel, Goretex) have precisely defined parameters, i.e., thickness, porosity, pore size, or curvature. In the case of polymer inclusion membranes (PIMs), formed by the pouring of a polymer, plasticizer, and carrier solution, the structure of the formed membrane differs, depending on the type and concentration of the substances. Therefore, it is necessary to examine the microstructure of the membrane material surface to determine basic parameters, such as the carrier distribution in the polymer matrix, or surface porosity and roughness. To this date, only a few studies [35,36,37,38,39,40,41,42] on the membrane morphology have been published. Amongst various techniques for examining surfaces, scanning electron microscopy (SEM) and atomic force microscopy (AFM) are the most useful and commonly used for this purpose.

Recently, PIMs have also been examined for their thermal stability [36,41,43]. For this purpose, they undergo a thermal analysis to determine thermal stability of polymers and to specify temperature ranges and thermal effects of their thermal degradation. The aim of this work was to check the possibility of separation and recovery of Zn(II) from mixtures of Zn(II)–Cd(II)–Ni(II) ions in aqueous solutions, using PIMs. For this purpose, alkyl derivatives of imidazole with small alkyl groups close to the donor nitrogen have influence on the complex formation, and the large alkyl substituent in the 1-position ensures the hydrophobicity of the molecule, selected as a carrier in PIMs. In the discussed case, 1-octylimidazole, 1-octyl-2-methylimidazole, 1-octyl-4-methylimidazole, and 1-octyl-2,4-dimethylimidazole were used as carriers. It was assumed that by different formation of the zinc, cadmium, and nickel ions complexes based on the above properties, it would be possible to diversify their properties, thus enabling their separation.

## 2. Experimental

### 2.1. Reagents

Analytical grade chemical reagents including zinc(II), cadmium(II), and nickel(II) nitrates; sulphuric acid; and tetramethylammonium hydroxide were purchased from (POCh, Gliwice, Poland). All aqueous solutions were prepared using analytical reagent grade chemicals and deionized water (conductivity, 0.10 μS/cm). Solutions of Zn(II) and Cd(II) were prepared by dissolving appropriate amounts of Zn(NO_3_)_2_·6H_2_O, Cd(NO_3_)_2_·4H_2_O, and Ni(NO_3_)_2_·6H_2_O in deionized water.

**Figure 1:**
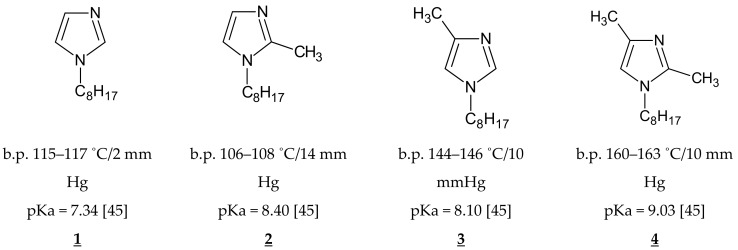
The chemical formula of alkylimidazole.

Analytical grade organic reagents, i.e., cellulose triacetate (CTA), o-nitrophenyl pentyl ether (o-NPPE), and dichloromethane (all from Sigma-Aldrich Company, Poznan, Poland) were used without further purification. The compounds 1-octylimidazole (**1**), 1-octyl-2-methylimidazole (**2**), 1-octyl-4-methylimidazole (**3**), and 1-octyl-2,4-dimethylimidazole (**4**) (Figure 1) were synthesized according to the procedure described in [44] by the alkylation of imidazole (Sigma-Aldrich Company, Poznan, Poland), 2-methylimidazole (Sigma-Aldrich Company, Poznan, Poland), 4-methylimidazole (Sigma-Aldrich Company, Poznan, Poland), and 2,4-dimethylimidazole (Sigma-Aldrich Company, Poznan, Poland) n-octyl bromide (ICP Industrial Product, Beer Sheva, Israel) in yields amounting to 78%, 65%, 63%, and 68%, respectively [45].

The carrier’s structure was confirmed by 1H NMR (400 MHz,), 13C NMR (100.6 MHz), and 15N NMR (40 MHz,) spectroscopy (Brucker, Hanau Germany).

**1**: ^1^H NMR, CDCl_3_, δ(ppm): 0.821 (t, 3H, C–CH_3_), 1.259 (m, 6H, C–(CH_2_)_3_–C), 1.671 (m, 6H, C–(CH_2_)_3_–C), 3.829 (t, 2H, CH_2_–N^1^), 7.378 (s, 1H, ^2^CH), 6.955 (s, 1H, ^4^CH), 6.819 (s, 1H, ^5^CH); ^13^C NMR, CDCl_3_, δ(ppm): 13.925, 22.804, 26.500, 28.948, 29.253, 31.699, 39.556, 46.712, 115.251, 129.353, 135.970; ^15^N NMR CDCl_3_, δ(ppm): 256.34, 173.47.

**2**: ^1^H NMR, CDCl_3_, δ(ppm): 0.781 (t, 3H, C–CH_3_), 1.808 (m, 6H, C–(CH_2_)_3_–C), 1.742 (m, 6H, C–(CH_2_)_3_–C), 3.823 (t, 2H, CH_2_–N^1^), 2.398 (s, 3H, ^2^C–CH_3_), 6.808 (s, 1H, ^5^CH), 7.005 (s, 1H, ^4^CH); ^13^C NMR, CDCl_3_, δ(ppm): 13.998, 22.554, 26.523, 28.991, 29.045, 31.045, 31.679, 32.025, 47.019, 118.740, 129.303, 137.019; ^15^N NMR CDCl_3_, δ(ppm): 255.13, 173.82.

**3**: ^1^H NMR, CDCl_3_, δ(ppm): 0.810 (t, 3H, C–CH_3_), 1.258 (m, 6H, C–(CH_2_)_3_–C), 1.691 (m, 6H, C–(CH_2_)_3_–C), 3.764 (t, 2H, CH_2_–N^1^), 2.249 (s, 3H, ^4^C–CH3), 6.733 (s, 1H, ^5^CH), 6.580 (s, 1H, ^2^CH); ^13^C NMR, CDCl_3_, δ(ppm): 13.937, 22.496, 26.463, 28.989, 29.083, 31.632, 38.556, 46.892, 117.961, 126.959, 136.522; ^15^N NMR CDCl_3_, δ(ppm): 256.68, 174.61.

**4**: ^1^H NMR, CDCl_3_, δ(ppm): 0.775 (t, 3H, C–CH_3_), 1.607 (m, 6H, C–(CH_2_)_3_–C), 1.945 (m, 6H, C–(CH_2_)_3_–C), 3.811 (t, 2H, CH_2_–N^1^), 2.249 (s, 3H, ^2^C–CH_3_), 6.642 (s, 1H, ^5^CH), 7.608 (s, 3H, ^4^C–CH_3_); ^13^C NMR, CDCl_3_, δ(ppm): 13.712, 22.414, 26.603, 27.882, 29.009, 30.925, 32.071, 39.556, 46.854, 47.262, 115.251, 128.969, 137.543; ^15^N NMR CDCl_3_, δ(ppm): 255.76, 172.63.

#### The Preparation and Characteristics of Polymer Inclusion Membranes

The concentration of the ion carrier in the membrane was 60%, since earlier studies [22,23,26,29,30,42,46] indicate that this concentration is optimum for that group of carriers.

The polymer membranes were prepared according to the procedure reported in our previous papers [22,23,26,29,30,42,46,47,48]. Organic solutions of the 35% support (CTA), the 60% ion carrier (**1**–**4**), and 5% the plasticizer (o-nitrophenylpentyl ether (o-NPPE)) solutions in dichloromethane were prepared. A portion of such a solution was poured into a membrane mold comprised of a 6.0 cm glass ring attached to a glass plate with CTA–dichloromethane glue. The organic solvent was allowed to evaporate overnight and the resulting membrane was separated from the glass plate by immersion in cold water.

The thickness of the PIM was measured using a digital micrometer (Panametrics^®^ Magna-Mike^®^ 8500 (San Diego, CA, USA)) with an accuracy of 0.1 µm. The thickness of a membrane was measured 10 times for each case and shown as the average value of these measurements, with the standard deviation below 1%. The thickness of membranes before and after transport was found to be the same. The average PIM thickness varied in the range 29–33 μm.

Each experiential point was repeated 4 times, i.e., membrane formed by immobilization, thickness measured, and transport parameters calculated. Experimental reproducibility was high with standard deviation below 1% of the measured values.

A surface characterization study of the polymer membranes was performed using an Atomic-force MultiMode Scanning Probe Microscope IIIa (AFM) (Digital Instruments Vecco Metrology Group, Santa Barbara, CA, USA) according to the procedure described in other papers [49,50,51]. Pore characterization was performed using the AFM image processing program NanoScope v.5.12, which enabled the calculation of two parameters: roughness (R_q_) and porosity (ε). The R_q_ parameter is the standard deviation of the z values within the box cursor and is calculated as:

(1) $$R_{q}=\sqrt{\frac{\sum {\left(z_{i}\right)}^{2}}{n}}$$

Additionally, the PIMs were characterized by scanning electron microscopy (SEM). SEM images of membranes were obtained with a Hitachi SU 3500 SEM/EDS (energy dispersive spectroscopy) microscope operated at 10.0 kV. The membranes were visualized in 5.0 × 5.0 μm images.

### 2.2. Transport Studies

Transport experiments were carried out in a permeation cell described in earlier papers [22,23,26,29,30,42,46,47,48]. The membrane film (surface area 4.9 cm^2^) was tightly clamped between two cell compartments. Both, the source phase and the receiving aqueous phase (45 cm^3^ each) were mechanically stirred at 600 rpm. Metal nitrates were used in the source phase, whereas the receiving phase was 0.5 M solution of sulphuric acid. The PIM transport experiments were carried out at 20 ± 0.2 °C. Small samples of the aqueous receiving phase were taken periodically from the sampling port equipped with a syringe and analyzed by atomic absorption spectroscopy (AAS 240FS Spectrometer, Agilent, Santa Clara, CA, USA) to determine zinc(II), cadmium(II), and nickel(II) concentrations. The pH of the source phase equal to 5.8 was kept constant using tetramethylammonium hydroxide.

The kinetics of transport across PIMs was described as a first-order process with respect to the metal-ion concentration [52] expressed by Equation (2):

(2) $$\ln\left(\frac{c}{c_{i}}\right)=-\mathrm{kt}$$

In order to calculate the value of k, ln(c/c_0_) versus time was plotted. The rate constant values for two independent transport experiments were averaged, and the standard deviation was calculated. The permeability coefficient (P) was calculated according to Equation (3):

(3) $$P=-\frac{V}{A}k$$

The initial flux (J_0_) was calculated as:

(4) $$J_{i}=P\cdot C_{i}$$

The selectivity ratio (S) was defined as the ratio of initial fluxes for M_1_ and M_2_ metal ions, respectively:

(5) $$S=J_{i,M1}/J_{i,M2}$$

In order to describe the efficiency of metal removal from the source phase, the recovery factor (RF) was calculated:

(6) $$\mathrm{RF}=\frac{c_{i}-c}{c_{i}}\cdot 100\%$$

The reported values correspond to the average values of three replicates, with deviations within 5%.

## 3. Results and Discussion

### 3.1. Membrane Characterization

**Figure 2:**
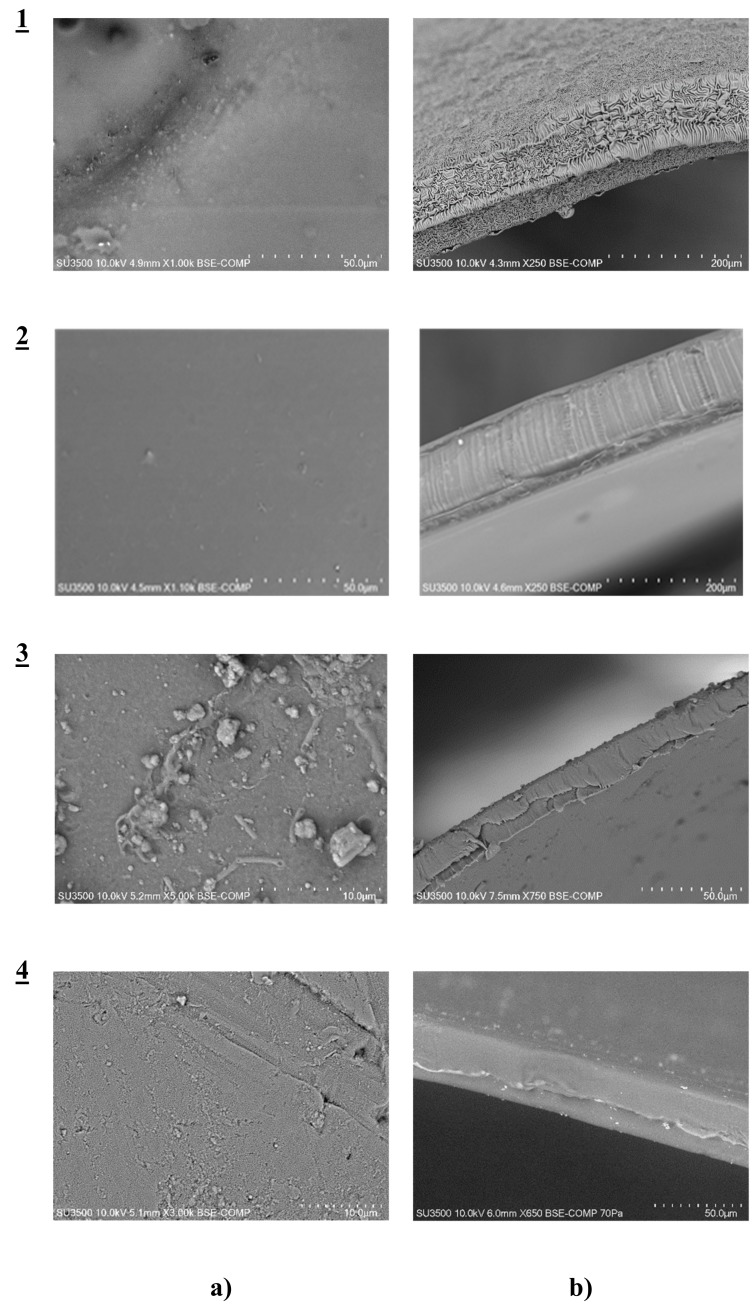
SEM images of polymer inclusion membranes (PIMs) containing 1-octylimidazole (**1**), 1-octyl-2-methylimidazole (**2**), 1-octyl-4-methylimidazole (**3**), and 1-octyl-2,4-dimethylimidazole (**4**) as a carrier: front view (**a**), cross-section along the diameter (**b**).

The SEM photomicrographs (Figure 2) showed that all membranes had dense and homogeneous structures. Moreover, the images showed visible roughness of film surfaces. Carriers could crystallize in the membrane and, for example, alkylimidazole molecules migrated to the membrane surface, causing its roughness and porosity.

The distribution of the carrier in the investigated membrane after the evaporation of dichloromethane was homogeneous on the entire surface. PIMs containing alkylimidazole (**1**–**4**) were dense and homogenous.

**Figure 3:**
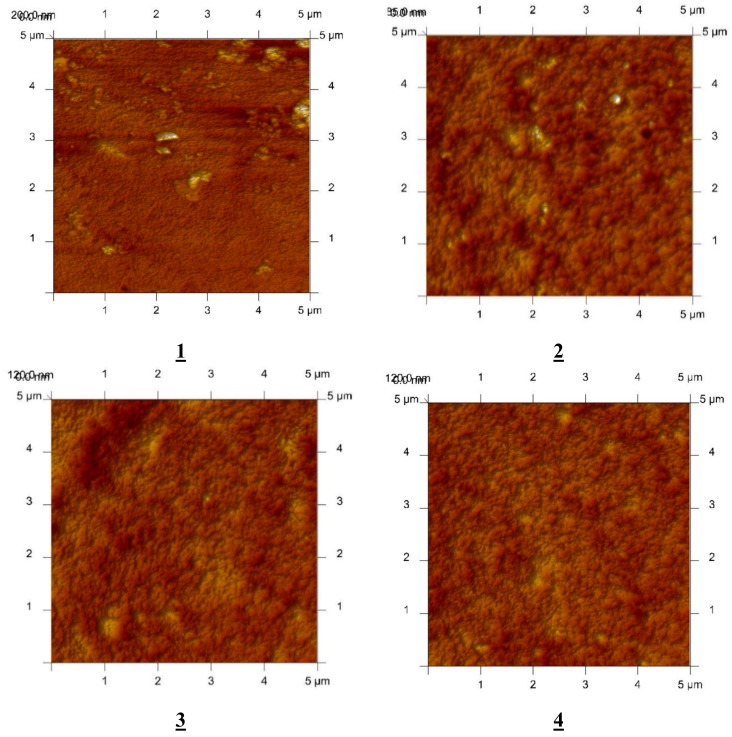
2D-view atomic force micrographs (AFMs) of PIMs with 1-octylimidazole (**1**), 1-octyl-2-methylimidazole (**2**), 1-octyl-4-methylimidazole (**3**), and 1-octyl-2,4-dimethylimidazole (**4**), at a 60% carrier concentration. Scan area: 5 × 5 µm.

An AFM image of the PIM doped with alkylimidazole (**1**–**4**) in two-dimensional forms is shown in Figure 3.

In the image of the PIM samples (Figure 3), clearly visible are elongated pores called also “cavity channels” (darker regions). Such a morphology of the membrane surface can be related to the crystallinity of the CTA. A wide network of pores of 5–25 nm in size is likely to be responsible for the improved transport performance of the membranes.

The roughness (R_q_) and effective pore sizes of the membrane were calculated using atomic force microscopy (AFM), and they are shown in Table 1 along with the data of membranes doped with 1-decyl-2-methylimidazole [30], 1-decyl-4-methylimidazole [26], and 1-decyl-2,4-dimethylimidazole [53].

The membrane’s tortuosity was determined from the dependence developed by Wolf and Strieder [54]:

(7) τ = 1− ln ε

As demonstrated in a number of papers [40,41,49,50,51], the microstructure of the membrane has an impact on the transport process. CTA membranes have porous structures, and the distribution of pores is nearly uniform (porosity 50%) [52]. The pores in a CTA matrix are filled with a plasticizer (o-NPPE) and the carrier. The carrier crystallizes inside the membrane, with the texture of the surface being relatively homogeneous, with different porosities and roughnesses. The roughness of a CTA membrane obtained by Tor et al. [51] equaled 14 nm, whereas the roughness of a CTA–o-NPPE membrane obtained by Radzyminska-Lenarcik and Ulewicz [47] equaled 6.8 nm. As seen in Table 1, the roughness of polymer inclusion membranes decrease in the sequence 3 > 2 > 4 > 1. For the tested carriers, no relationship was observed between the roughness of the membrane and the molar intrinsic volumes of the tested carriers, as was previously observed when the derivatives of imidazole azothiacrown ethers [49] were used. Nevertheless, the membranes with 1-octyl-alkyl derivatives of imidazole had lower molar intrinsic volumes and showed lower roughness than membranes with 1-decyl-alkyl derivatives of imidazole used in papers [26,30,53], which had larger molar intrinsic volumes. The roughness values determined for polymer inclusion membranes with carriers **1**–**4** were insignificantly higher than for the commercial di-(2-ethylhexyl) phosphoric acid (D2EHPA) carrier (4.7 nm) used by Salazar-Alvarez [50].

### 3.2. Transport Zn(II), Cd(II), and Ni(II) across PIMs

Findings on the transport of non-ferrous metal ions from equimolar nitrate solutions, across PIMs containing 1-octylimidazole (**1**), 1-octyl-2-methylimidazole (**2**), 1-octyl-4-methylimidazole (**3**), and 1-octyl-2,4-dimethylimidazole (**4**) as ion carriers are discussed below. The studies were carried out from single- or multi-component solutions, containing metals each at a concentration of 0.001 M. The initial flux values and selectivity coefficients for the metal ion transport across the PIMs are shown in Table 2.

These form electrically neutral ion pairs with anions that penetrate the membrane in the co-transport:

(8) $$M^{2+}\;+\;L\;+\;2A‾\;↔\;\left(\mathrm{ML}\right)A_{2}$$

where M^2+^ is the metal ion, L is the carrier, and A‾ is the co-transported anion.

The stability constants values of Zn(II), Cd(II), and Ni(II) complexes with the investigated carriers are summarized in Table 3.

It follows from the data shown in Table 2 that the initial flux value for Zn(II) ion transport from a unary solution is higher than that from multi-component solutions. The initial flux values were the highest for membrane doped with **4**. For a ternary mixture (solution V), the initial fluxes of metal ions transported across PIMs containing all carriers (**1**–**4**) decreased in the following order: Zn(II) > Cd(II) > Ni(II). Selectivity coefficients (S) Zn(II)/Cd(II) were approximately equal to 1.5 for PIMs with carriers **1**–**3** and equal to 2.8 for PIMs doped with **4**. Selectivity coefficients (S) Cd(II)/Ni(II) increased in order **1** < **2** < **3** < **4** from 3.5 (**1**) up to 23.5 (**4**). Very high selectivity coefficients Zn(II)/Ni(II) were obtained when there was no cadmium in the mixture (solution IV), especially in the case of the carrier **4**. Both the initial fluxes and selectivity coefficient values depended on the carrier used. This was caused by differences in both the basicity of the carriers and the stability constants of their complexes with metal ions. Octyl substituents at position 1 of the imidazole ring distinctly affected the hydrophobic properties of the molecule. An additional two methyl substituents at positions 2 and 4 of the imidazole ring (due to the induction effect) were found to increase basicity by one order of magnitude, compared with that of 1-octylimidazole [45] (Figure 1). The strongest base was 1-octyl-2,4-dimethylimidazole (**4**) [45].

As we can see from Table 2, using 1-octyl-2,4-dimethylimidazole (**4**) allowed us to obtain the highest value of initial flux transport of zinc(II) ions (28 µmol/m^2^∙s) from the solution and the highest coefficient of separation in relation to Ni(II) and Cd(II) ions. Zinc(II) ions are selectively separated from chloride water solutions in the presence of Cd(II), Co(II), Cu(II), and Ni(II) ions in the process of transport through a PIM containing D2EHPA. The best Zn(II) separation selectivity compared to the other ions was obtained at pH ≤ 2.0. The ion transport rate decreased in the following order: Zn(II) > Cu(II) > Co(II) > Ni(II) > Cd(II). The Zn(II)/Cd(II), Zn(II)/Ni(II), Zn(II)/Co(II), and Zn(II)/Cu(II) selectivity ratios equaled 879, 24.4, 9.90, and 6.50, respectively [63,64], whereas using Cyanex 301 and Cyanex 302 enabled the separation of Pb(II) ions from Zn(II) and Cd(II) ions. The Pb/Cd and Pb/Zn separation factors for Cyanex 301 were 1.7 and 2.8, and for Cyanex 302, 1.9 and 4.4, respectively [65].

The use of alkylimidazoles **1**–**4** gave a better effect of the separation of Zn(II) and Ni(II) ions than using the polymer inclusion membrane with β-cyclodextrin [66], PNP-16-crown-6-derivatives [67], and tri-n-octylamine [68].

Separation of zinc(II) from cobalt(II) ions was possible using supported liquid membrane with DP-8R (di(2-ethylhexyl) phosphoric acid) as a carrier. Using 0.5 M sulphuric acid as receiving phase, with the increase of the initial DP-8R concentration (5–30%), the value of permeability of Zn(II) increased from 2.8 to 4.5∙10^−3^ cm/s [69].

Additionally, the transport of Zn(II) ions across the supported liquid membrane with TDDA (tri-n-dodecylamine) was possible. The maximum initial flux of Zn(II) (almost 10∙10^−11^ mol/m^2^∙s) was achieved at 0.80 mol/dm^3^ of TDDA in the membrane phase and 2.0 mol/dm^3^ of HCl and NaOH in feed and receiving solution, respectively [70]. Therefore, the initial flux of Zn(II) ion transport across the liquid membrane with TDDA was lower than using the investigated alkylimidazoles **1**–**4**.

Ion carriers embedded in the membrane interacted with metal ions in the diffusion-controlled water phase to form complexes.

As seen in Table 3, the stability constants for 1-octylimidazole (**1**) complexes with all metal ions were the highest. The presence of the methyl substituent at positions 2 or 4 of the imidazole ring hindered the formation of each metal complex (steric effect). The stability constants of Zn(II), Cd(II), and Ni(II) complexes increased in the following order: Zn(II) > Cd(II) > Ni(II) (Table 3). The steric effect differentiated the complexing properties of the metal ions tested, so it was possible to separate them.

In aqueous solutions, cations of Zn(II), Cd(II), and Ni(II) existed in the form of octahedral aqua complexes (M(H_2_O)_6_)^2+^. In the case of Zn(II) and Cd(II) ions, owing to the effect of ligands (extractants), octahedral aqua complexes of certain cations tended to change their coordination number (c.n.) from 6 to 4, and they changed their coordination sphere into square flat or deformed tetrahedron, depending on the structure of their d-electron layer. The process is illustrated by Equation (9).

(9) $${\left(M{\left(H_{2}O\right)}_{6-n}L_{n}\right)}^{2+}\;↔\;{\left(M{\left(H_{2}O\right)}_{4-n}L_{n}\right)}^{2+}+\;2H_{2}O\;\;{\left(M{\left(H_{2}O\right)}_{6-n+1}L_{n-1}\right)}^{2+}\;+\;L\;↔\;{\left(M{\left(H_{2}O\right)}_{4-n}L_{n}\right)}^{2+}\;+\;3H_{2}O$$

The steric effect of the substituent at position 2 or 4 decreases the stability constants of octahedral complexes of all the metals studied, although it does not hinder the formation of tetrahedral species [29,30,53,55,58,59,60,61,62]. The formation of tetrahedral complexes with 1-alkylimidazoles has been proven for the Zn(II) and Cd(II) ions [42,56,59,71]. The Zn(II) ions were transported across PIMs most effectively.

The Ni(II) ions most of all formed 6-coordination complexes because they have a rigid octahedral structure that is hard to deform. This may be the reason for their small transport.

It seems (compare Table 2 and Table 3) that the initial flux values corresponded to those of the stability constants of the complexes.

### 3.3. Recovery of Metal

**Figure 4:**
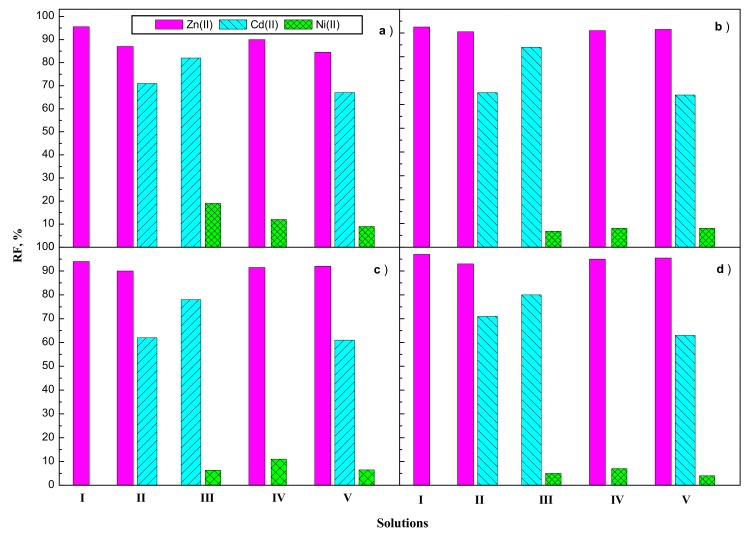
Recovery factors (RF) of Zn(II), Cd(II), and Ni(II) competitive transport across PIM doped alkylimidazoles **1** (**a**), **2** (**b**), **3** (**c**), and **4** (**d**).

The recovery factor (RF) of Zn(II) transport across PIM doped alkylimidazoles (**1**–**4**) from equimolar nitrate solutions of Zn(II), Cd(II), and Ni(II) into 0.5 M H_2_SO_4_ is presented in Figure 4. The RF values depended on the carrier in PIMs used.

The highest recovery factors (RF) were found for Zn(II) ions for unary solution for PIM doped **4** (97%). The RF values of Zn(II) ions from binary Zn(II)–Cd(II) solutions (solution II) increased in the following order: **1** < **2** = **3** < **4**. For the Zn(II)–Ni(II) mixture (solution IV) the RF values were almost the same (c.a. 90–91%) in the case of **1**–**3** carriers, but for carrier **4,** RF for the Zn–Ni mixture increased to 95%. For carrier **4**, in the case of a ternary Zn(II)–Cd(II)–Ni(II)-mixture, the RF for Zn(II), Cd(II), and Ni(II) were 95.5, 63, and 4 percent, respectively.

The lowest RF values were obtained for Ni(II) ions, which were the slowest transported by this type of membrane. Practically, Ni(II) ions remained in the feeding phase.

### 3.4. Membrane Diffusion Coefficients of Zn(II), Cd(II), and Ni(II) Complexes with Alkylimidazole (**1**–**4**)

**Figure 5:**
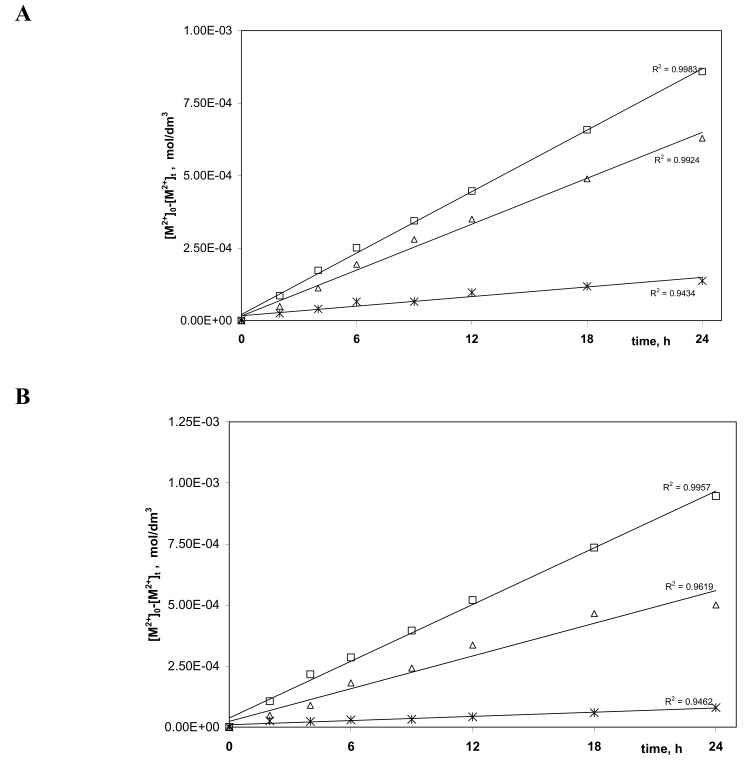

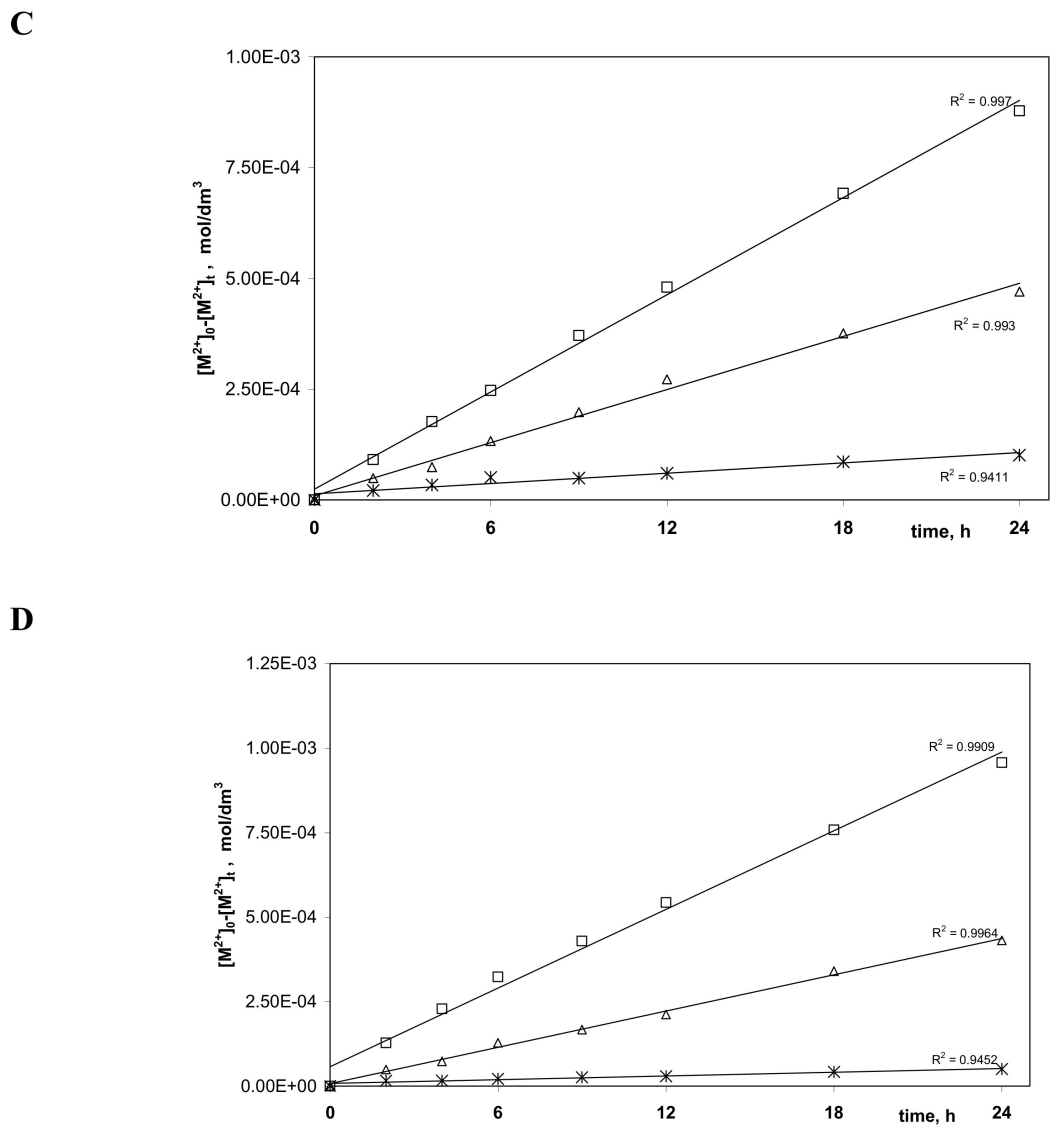
Relation of [M^2+^]_0-_[M^2+^]_t_ plotted vs. time for Zn(II) (□), Cd(II) (∆), and Ni(II) (*), transport across PIMs doped with 1-octylimdazole (**A**), 1-octyl-2-methylimidazole (**B**), 1-octyl-4-methylimidazole (**C**) and 1-octyl-2,4-methylimidazole (**D**), respectively.

In Figure 5A–D, the correlation graphs (M^2+^)_0–_(M^2+^)_t_ versus time of metal ions transport across PIM doped with alkylimidazole (**1**–**4**) are presented.

The diffusion coefficient of M(II) (D_o_)was calculated from the equation:

(10) $$D_{o}=d_{o}/\Delta_{o}$$

where d_o_ is the thickness of the membrane (0.003 cm) and Δ_o_ could be evaluated by plotting (M^2+^)_0-_(M^2+^)_t_ vs time.

The corrected (normalized) membrane diffusion coefficient D_o,n_ [50], which considers the morphological features inside the membrane (ε—porosity and τ—tortuosity), was calculated from the following equation:

(11) $$D_{o,n}={\;D}_{o}\cdot \left(\varepsilon \;/\;\tau \right)$$

The obtained values of diffusion coefficients are presented in Table 4.

Values of diffusion coefficients determined in this study are comparable with those presented in the literature data for different membranes, which are in the range of 10^−12^ to 10^−6^ cm^2^/s, and show that the limiting step of the process is the transfer of metal complexes across the membrane barrier. The value of the diffusion coefficient of M(II)–carrier species of 4.15∙10^−11^–2.04∙10^−8^ cm^2^/s is smaller than the value of 1.5∙10^−7^ cm^2^/s reported for the Pb(II) complex with the di-(2-ethylhexyl) phosphoric acid (D2EHPA) in PIMs reported by Salazar-Alvarez et al. [50].

The values of normalized diffusion coefficients (considering membrane porosity and tortuosity) of M(II)–carrier complexes, obtained in the process of transport across PIMs containing alkylimidazole (**1**–**4**) from the equimolar Zn(II)–Cd(II)–Ni(II) solution, are in the range 4.53∙10^−12^–2.11∙10^−9^ cm^2^/s.

Thus, the rate of transport of non-ferrous metal ions across PIMs doped with alkylimidazole (**1**–**4**) is determined by the diffusion rate of the M(II)–carrier complexes across the membrane.

## 4. Conclusions

Zinc(II) ions can be effectively separated from equimolar aqueous solutions of zinc, cadmium, and nickel nitrates by using the transport across polymer inclusion membranes doped with 1-octylimidazole (**1**), 1-octyl-2-methylimidazole (**2**), 1-octyl-4-methylimidazole (**3**), and 1-octyl-2,4-dimethylimidazole (**4**). The initial fluxes of metal ion transport decreased in the following order: Zn(II) > Cd(II) > Ni(II). The transport rate of the metal ions across PIMs was determined by the diffusion rate of the M(II)–carrier complexes across the membrane.

The best result achieved for Zn(II) removal after 24 h was 95.5% for the ternary Zn(II)–Cd(II)–Ni(II) solution for PIM doped 1-octyl-2,4-dimethylimidazole (**4**). For this membrane, the separation coefficients for Zn(II)/Cd(II), Zn(II)/Ni(II), and Cd(II)/Ni(II) were 2.8, 104.5, and 23.5, respectively. This effective separation was possibly achieved due to the steric effect induced by the presence of the methyl substituent in a direct vicinity of the donor nitrogen atom in the carrier molecule. It differentiates the process of the complex formation, particularly for Zn(II) and Ni(II). Carriers whose particles have a steric effect can also be used to separate Co(II) ions, because this cation has the ability to change the coordination sphere from octahedron to tetrahedron.

## Figures and Tables

**Table 1:** AFM characterization parameters for PIMs doped with alkylimidazoles. CTA–o-NPPE is cellulose triacetate–o-nitrophenyl pentyl ether.

Carrier in the CTA–o-NPPE Membrane	Effective Pore Size (µm)	Tortuosity	Roughness (R_q_) (nm)	Ref.
1-decyl-2-methylimidazole	0.057	2.81	7.2	[30]
1-decyl-4-methylimidazole	0.060	2.85	6.7	[26]
1-decyl-2,4-dimethylimidazole	0.065	2.45	5.8	[53]
1-octylimidazole (**1**)	0.051	2.15	5.4	this work
1-octyl-2-methylimidazole (**2**)	0.054	2.38	6.1	this work
1-octyl-4-methylimidazole (**3**)	0.058	2.75	6.5	this work
1-octyl-2,4-dimethylimidazole (**4**)	0.062	2.60	6.0	this work

**Table 2:** Initial fluxes (J_0_), selectivity order, and selectivity coefficients (S) for the competitive transport of Zn(II), Cd(II), and Ni(II) ions across PIMs doped with alkylimidazoles **1**–**4.**

Carrier	Solutions	Metal Ions	J_0_ (µmol/m^2^∙s)	Selectivity Order Selectivity Coefficients S_Zn(II)/M(II)_
**1**	**I**	Zn(II)	16.32	-
	**II**	Zn(II) Cd(II)	11.30 7.96	Zn(II) > Cd(II) 1.4
	**III**	Cd(II) Ni(II)	8.56 2.42	Cd(II) > Ni(II) 3.5
	**IV**	Zn(II Ni(II)	10.83 1.07	Zn(II) > Ni(II) 10.1
	**V**	Zn(II) Cd(II) Ni(II)	10.76 6.61 1.18	Zn(II) > Cd(II) > Ni(II) S_Zn(II)/Cd(II)_—1.6, S_Zn(II)/Ni(II)_—9.1
**2**	**I**	Zn(II)	12.45	-
	**II**	Zn(II) Cd(II)	10.13 6.24	Zn(II) > Cd(II) 1.6
	**III**	Cd(II) Ni(II)	7.16 0.45	Cd(II) > Ni(II) 15.9
	**IV**	Zn(II) Ni(II)	9.82 0.43	Zn(II) > Ni(II) 22.7
	**V**	Zn(II) Cd(II) Ni(II)	8.49 5.83 0.30	Zn(II) > Cd(II) > Ni(II) S_Zn(II)/Cd(II)_—1.5, S_Zn(II)/Ni(II)_—18.0
**3**	**I**	Zn(II)	11.68	-
	**II**	Zn(II) Cd(II)	9.53 7.01	Zn(II) > Cd(II) 1.4
	**III**	Cd(II) Ni(II)	6.92 0.35	Cd(II) > Ni(II) 19.8
	**IV**	Zn(II) Ni(II)	10.02 0.29	Zn(II) > Ni(II) 34.6
	**V**	Zn(II) Cd(II) Ni(II)	8.97 5.61 0.37	Zn(II) > Cd(II) > Ni(II) S_Zn(II)/Cd(II)_—1.6, S_Zn(II)/Ni(II)_—22.9
**4**	**I**	Zn(II)	28.13	-
	**II**	Zn(II) Cd(II)	26.08 9.24	Zn(II) > Cd(II) 2.8
	**III**	Cd(II) Ni(II)	8.47 0.36	Cd(II) > Ni(II) 23.5
	**IV**	Zn(II) Ni(II)	27.12 0.25	Zn(II) > Ni(II) 104.5
	**V**	Zn(II) Cd(II) Ni(II)	25.44 7.62 0.29	Zn(II) > Cd(II) > Ni(II) S_Zn(II)/Cd(II)_—3.3, S_Zn(II)/Ni(II)_—87.7

**Table 3:** Comparison of the stability constants β_n_ of Zn(II), Cd(II), and Ni(II) complexes with alkylimidazole **1**–**4**, at 25 °C, ionic strength 0.5 M (KNO_3_).

Ligand/Carrier	Metal Ion	log β_1_	log β_2_	log β_3_	log β_4_	Ref.
**1**	**Zn(II)**	2.73	4.15	6.64	10.18	[55]
	**Cd(II)**	1.95	2.81	4.00	5.20	[56]
	**Ni(II)**	0.79	3.90	6.20	8.10	[57]
**2**	**Zn(II)**	1.96	4.45	6.80	9.10	[58]
	**Cd(II)**	1.03	2.20	3.45	5.05	[58]
	**Ni(II)**	0.11	0.47	1.19	2.63	[59]
**3**	**Zn(II)**	2.04	3.50	6.20	6.90	[60]
	**Cd(II)**	1.26	2.20	3.93	5.11	[61]
	**Ni(II)**	0.69	1.04	2.00	2.92	[61]
**4**	**Zn(II)**	1.65	2.17	4.48	6.39	[62]
	**Cd(II)**	1.17	2.53	4.21	5.68	[62]
	**Ni(II)**	0.09	0.22	1.11	2.05	[62]

**Table 4:** Diffusion coefficients normalized for competitive transport of Zn(II), Cd(II), and Ni(II) ions through PIMs doped with alkylimidazole (**1**–**4**).

Carrier	Metal Ion	Δ_o_ (s/m)	D_o_ (cm^2^/s)	D_o,n (_cm^2^/s)
**1**	Zn(II)	107.25	2.04·10^−8^	2.11·10^−9^
	Cd(II)	108.49	7.16·10^−9^	7.40·10^−10^
	Ni(II)	1010.11	4.39·10^−11^	4.53·10^−12^
**2**	Zn(II)	106.14	1.92·10^−8^	2.01·10^−9^
	Cd(II)	107.63	6.76·10^−9^	7.14·10^−10^
	Ni(II)	1010.65	4.15·10^−11^	4.27·10^−12^
**3**	Zn(II)	106.67	1.72·10^−8^	1.98·10^−9^
	Cd(II)	108.02	6.34·10^−9^	6.75·10^−10^
	Ni(II)	1010.50	4.59·10^−11^	4.83·10^−12^
**4**	Zn(II)	106.08	1.53·10^−8^	1.74·10^−9^
	Cd(II)	107.15	6.06·10^−9^	6.58·10^−10^
	Ni(II)	1010.61	4.72·10^−11^	4.91·10^−12^
